# Role of long noncoding RNAs in diabetes-associated peripheral arterial disease

**DOI:** 10.1186/s12933-024-02327-7

**Published:** 2024-07-24

**Authors:** Alonso Tapia, Xuejing Liu, Naseeb Kaur Malhi, Dongqiang Yuan, Muxi Chen, Kevin W. Southerland, Yingjun Luo, Zhen Bouman Chen

**Affiliations:** 1 Irell and Manella Graduate School of Biological Sciences, City of Hope, Duarte, CA 91010 USA; 2 Department of Diabetes Complications and Metabolism, Arthur Riggs Diabetes and Metabolism Research Institute, City of Hope, Duarte, CA USA; 3 Division of Vascular and Endovascular Surgery, Department of Surgery, Duke University Medical Center, Durham, NC 27710 USA

**Keywords:** Diabetes mellitus (DM), Long non-coding RNA (lncRNA), Endothelial cells (ECs), Vascular smooth muscle cells (VSMCs), Macrophages (MΦs), Atherosclerosis, Peripheral arterial disease (PAD)

## Abstract

Diabetes mellitus (DM) is a metabolic disease that heightens the risks of many vascular complications, including peripheral arterial disease (PAD). Various types of cells, including but not limited to endothelial cells (ECs), vascular smooth muscle cells (VSMCs), and macrophages (MΦs), play crucial roles in the pathogenesis of DM-PAD. Long non-coding RNAs (lncRNAs) are epigenetic regulators that play important roles in cellular function, and their dysregulation in DM can contribute to PAD. This review focuses on the developing field of lncRNAs and their emerging roles in linking DM and PAD. We review the studies investigating the role of lncRNAs in crucial cellular processes contributing to DM-PAD, including those in ECs, VSMCs, and MΦ. By examining the intricate molecular landscape governed by lncRNAs in these relevant cell types, we hope to shed light on the roles of lncRNAs in EC dysfunction, inflammatory responses, and vascular remodeling contributing to DM-PAD. Additionally, we provide an overview of the research approach and methodologies, from identifying disease-relevant lncRNAs to characterizing their molecular and cellular functions in the context of DM-PAD. We also discuss the potential of leveraging lncRNAs in the diagnosis and therapeutics for DM-PAD. Collectively, this review provides a summary of lncRNA-regulated cell functions contributing to DM-PAD and highlights the translational potential of leveraging lncRNA biology to tackle this increasingly prevalent and complex disease.

**Graphical Abstract:**

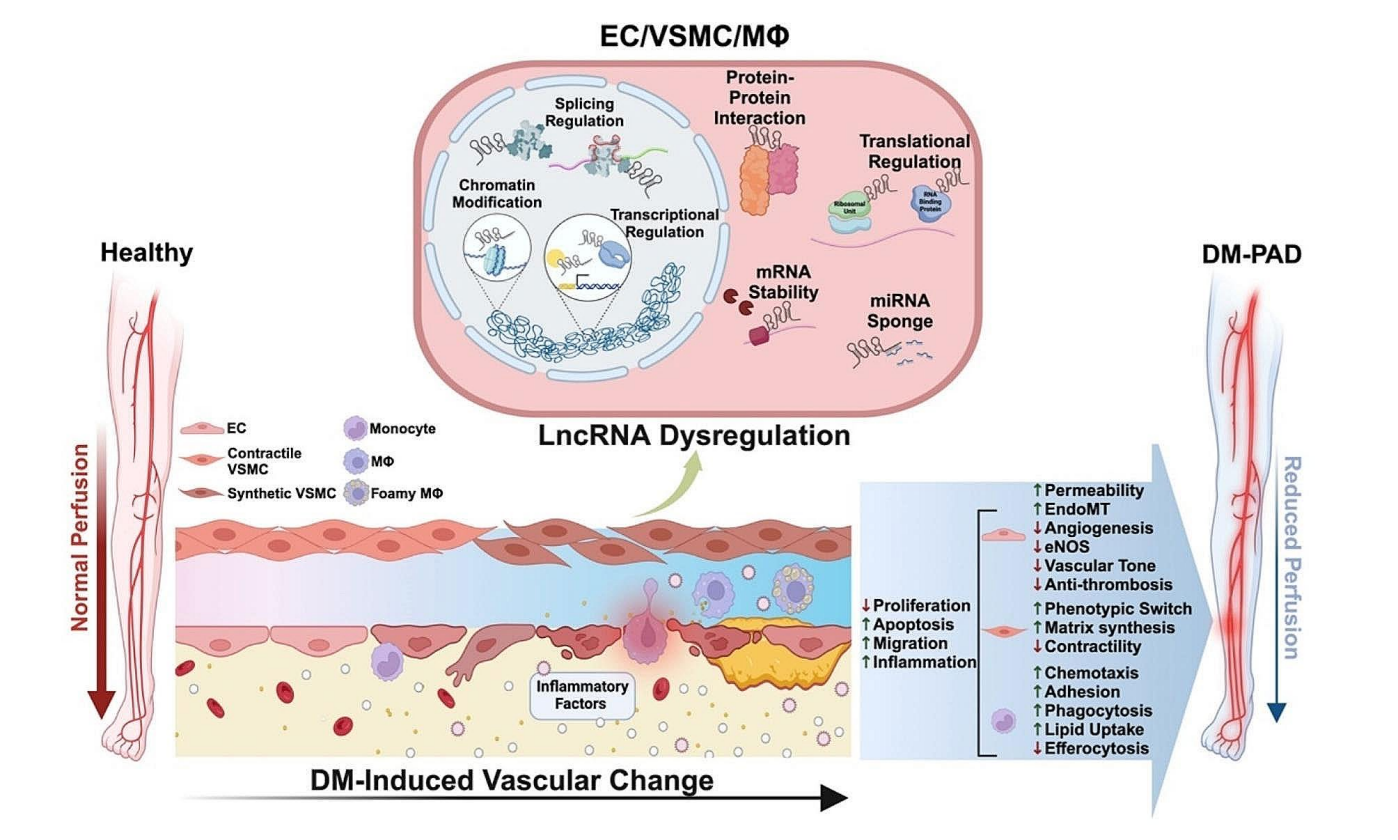

## Introduction

Diabetes or diabetes mellitus (DM) is a disease characterized by the poor control of blood glucose levels and is broadly divided into Type 1 (T1D), Type 2 (T2D), and gestational diabetes, all resulting in hyperglycemia. In 2021, the CDC estimated global diabetes prevalence in individuals ranging from 20 to 70 years old was 10.5% (half a billion people), which is predicted to rise to 12.2% (783 million people) by 2045 [1]. The financial burden attributed to diabetes-related expenditures is estimated to be over a trillion USD by 2045 [2], posing a daunting socio-economic burden. Patients with DM are at high risk for various cardiovascular diseases (CVD), e.g., coronary artery disease (CAD), stroke, and peripheral artery disease (PAD), which are the leading causes of DM-related mortality and morbidity. Specifically, PAD occurs in patients with DM at a two- to four-fold higher incidence than in individuals without DM [3]. While sharing some etiology with CAD and stroke, PAD, defined by reduced blood flow as a result of the narrowing of arteries to the limbs, has its unique pathogenesis. Although PAD can also affect the upper limbs, it is much more prevalent in lower limbs.

Concomitant DM-PAD represents a significant threat to both life and limb. There are 185,000 amputations in the US each year, and more than half are attributable to DM-PAD [4, 5]. DM-PAD carries a risk of limb loss four times higher than the national average [6, 7]. The primary therapy for limb preservation in this patient cohort is revascularization [8]. However, the efficacy of revascularization in this patient cohort is severely hampered by two biological hurdles that are closely associated with DM: (1) severe vessel calcification and (2) microvascular disease. Significant medial artery calcification is a common histologic feature among people with diabetes and is associated with adverse outcomes [9–11]. A myriad of surgical techniques, including atherectomy, lithotripsy, and covered stents, have been developed to treat calcium-laden atherosclerotic lesions. However, none of these have improved clinical outcomes [12]. Microvascular disease, defined as occlusive lesions in blood vessels ≤ 100 μm in diameter, is strongly associated with diabetes [13] and increases the risk of amputation four-fold [14]. Currently, there are no surgical or medical therapies for microvascular disease. Given the aging population and growing incidence of diabetes, there is a critical, unmet need to deepen our understanding of the pathobiology of both DM and PAD and to leverage that knowledge to develop novel therapies to improve PAD outcomes.

Many risk factors associated with DM, e.g., hyperglycemia, hyperlipidemia, and hypertension, can contribute to atherosclerosis, characterized by a buildup of plaque in the inner arterial wall resulting in restricted blood flow [15, 16]. Atherosclerosis begins with dysfunctional endothelial cells (ECs), which, in a classical model, recruit monocytes to attach, migrate, and differentiate into macrophages (MΦs). After engulfing low-density lipoprotein (LDL) cholesterol, MΦs become foam cells in the vascular wall, a hallmark of atherosclerotic lesions [16]. Other MΦs polarize to an inflammatory state, which, along with ECs, secrete platelet-derived growth factors (PDGFs), vascular endothelial growth factors (VEGF), and other inflammatory cytokines, resulting in the increased proliferation of vascular smooth muscle cells (VSMCs), which can undergo dedifferentiation and phenotypic switching towards myofibroblast- or an osteoblast-like phenotype [16, 17]. These cells secrete collagen and other extracellular matrix (ECM) components, leading to lesion progression, calcification, and fibrosis of the arterial wall. At the later stage, prolonged exposure to an inflammatory milieu results in apoptosis of ECs, VSMCs, and MΦ, forming a necrotic core in the atherosclerotic lesion. In a diabetic setting, where patients are atheroprone, the capability of MΦs to participate in efferocytosis, the process of removing diseased and dying cells through phagocytosis, is often hampered [18]. Without inflammation resolution, the plaque can destabilize and rupture, causing thromboembolism, inadequate oxygenation, and nutrient delivery in the periphery.

To aggravate these detrimental alterations, the microvascular ECs in the diabetic vessels can have impaired angiogenic and wound healing capacity, failing to form adequate collateral vessels to alleviate the tissue ischemia. Compensatory angiogenesis is supported by tissue-resident MΦ recruitment, which secretes pro-angiogenic factors such as VEGF and recruits other support cells, including monocytes and neutrophils. However, given the dominance of pro-inflammatory MΦ due to the DM milieu, this restorative mechanism is often insufficient to overcome the ischemia [19, 20]. The compromised tissue perfusion and ischemia in the limbs and extremities can lead to intermittent claudication (IC), manifesting in cramping and pain, a common symptom of PAD. When these conditions continue to worsen, PAD can progress to critical limb tissue ischemia (CLTI), the most severe form of PAD [21]. CLTI presents as resting pain, severe tissue ischemia, and impaired wound healing and can be coupled with peripheral sensitive neuropathy, a condition also more common in patients with diabetes [22]. Concomitant DM-PAD can cause the formation of blisters/ulcers from imbalanced weight support, numbness even in the presence of injury, and insufficient wound healing to resolve skin wounds, all of which place patients at higher risk of infections [23]. These detrimental factors work synergistically to further drive tissue death and potential limb amputation as a consequence of diabetic foot.

In the past decades, the DM-PAD field has primarily focused on dissecting the role of proteome and the related cellular pathways in regulating various cellular processes contributing to this ischemic disease. Recent technological advances in human transcriptome profiling have enabled the thorough investigation of the non-coding genome, including long non-coding RNAs (lncRNAs). Like messenger RNAs (mRNAs), most lncRNAs are transcribed by RNA polymerase II, 7-methyl guanosine (m^7^G)-capped at the 5′ ends and polyadenylated at the 3′ end [24, 25]. Moreover, lncRNAs display distinct features in their transcription, processing, and subcellular localizations, which dictate their modes of action and cellular functions. Relative to protein-coding mRNAs, lncRNAs display higher cell-type specificity and exert varied functions in different cell types. While there are multiple ways to classify lncRNAs, one is by their genomic locus and orientation to nearby protein-coding genes [26–28]. This results in four classes: intergenic, intronic, sense, and antisense lncRNA. Early studies focused on long intergenic non-coding RNAs (lincRNAs), whose transcriptional units do not overlap with nearby protein-coding genes. Intronic lncRNAs initiate from the intron of a protein-coding gene, while sense lncRNAs are transcribed from the sense strand of a protein-coding gene and its exons, overlapping with part of protein-coding genes. In contrast, antisense lncRNAs are transcribed from the antisense strand of protein-coding genes, usually overlapping with protein-coding exons.

LncRNAs can also be classified based on their mechanism of action, i.e., *cis*- and *trans*-acting [29–31]. While *cis-*acting lncRNAs exert regulatory control over the transcription within the same or adjacent loci, *trans-*acting lncRNAs exhibit their regulatory roles on distal targets, the mechanism of which appears much more diverse and less characterized. However, some lncRNAs can follow both *cis*- and *trans*-acting modes of action. To add to the complex biology of lncRNAs, promoter regions and pseudogenes can also give rise to lncRNAs. A significant proportion of lncRNAs are transcribed from enhancers, the DNA-regulatory elements that activate transcription to higher levels than those in their absence [32–35]. The genomic loci of these lncRNAs are typically associated with enhancer histone hallmarks or bound by transcription factors (TFs), also termed super-enhancers (SEs) [36, 37]. Previous studies, including ours, reported that lncRNAs derived from SE regions show strong cell type specificity and participate in transcription regulation of cell identity, fate, and stress responses (i.e., angiogenesis and proliferation) [38–41]. Understanding the classification of lncRNAs can aid in a systematic, comprehensive approach to their diverse roles and regulatory mechanisms in diseases like DM-PAD, which can be leveraged to design new avenues for disease management, treatment, and intervention.

In this review, we summarize the existing literature on lncRNA regulation of the dominant vascular cell types contributing to DM-PAD, using selective lncRNAs that have been extensively studied as examples. While atherosclerosis is the main cause of PAD and impaired angiogenesis is a primary contributor to PAD, several excellent review articles have discussed the role of lncRNA in atherosclerosis and angiogenesis [42–50]. Herein, we place an emphasis on lncRNAs that can serve as missing links between DM and PAD. We then provide suggestions for experimental approaches to study lncRNAs in DM-PAD. Finally, we discuss the potential of exploiting lncRNAs as diagnostic and therapeutic targets for treating DM-PAD. Collectively, we hope to provide an overview of the emerging role of lncRNA in DM-PAD that can spur future exploration in this research area.

## LncRNAs in endothelial cells (ECs)

The endothelium constitutes the critical interface between tissues and circulation. In physiological conditions, ECs are quiescent, exerting anti-inflammatory and anti-thrombotic properties. In the diabetic milieu, ECs become activated and upregulate adhesion molecules such as vascular cell adhesion molecule-1 (VCAM-1) and monocyte chemoattractant protein (MCP1, coded by *CCL2*), recruiting leukocytes, a key step of vascular inflammation. Diabetic stress also leads to decreased nitric oxide (NO) bioavailability and impaired NO-mediated endothelium-dependent vasodilation, the hallmark of EC dysfunction. Increased oxidative stress is another detrimental factor due to diabetic stress, resulting from activation of NADPH oxidases (NOX), mitochondrial dysfunction, and endothelial NO synthase (eNOS) uncoupling [51–53]. The chronic elevation of glucose, pro-inflammatory cytokines (e.g., tumor necrosis alpha (TNF-α), interleukin-1 beta (IL-1β)), and oxidative stress can induce endothelial-mesenchymal transition (EndoMT), characterized by downregulation of the endothelial lineage markers (e.g., cadherin-5, and eNOS) and upregulation of the mesenchymal markers (e.g., alpha-smooth muscle actin (α-SMA), type I collagen, and fibronectin 1 (FN1) [54, 55]. EC apoptosis can occur at the late stage of PAD, resulting in plaque erosion and thrombosis. While these pathogenic processes can be shared by both large and small vessels, microvascular EC dysfunction can manifest as reduced angiogenic capacity, a crucial aspect of the pathogenesis of PAD [56]. An increasing list of lncRNAs has been shown to regulate EC function, as summarized by several excellent reviews [42, 50, 57–65]. Below, we highlight the reported roles of several lncRNAs that may serve as molecular links connecting DM and PAD.

Metastasis Associated Lung Adenocarcinoma Transcript 1 (MALAT1) is one of the first identified and most extensively studied lncRNAs implicated in numerous diseases, including DM, CAD, and PAD [66–68]. It is ubiquitously expressed, highly conserved across mammalian species [69], and primarily localized to the nucleus [70, 71]. *MALAT1* is encoded within Chromosome 11q13 in humans and Chromosome 19qA in mice. In ECs, MALAT1 regulation seems context-dependent with different modes of action. For example, MALAT1 is increased in streptozotocin (STZ)-treated diabetic mice, and high-glucose (HG) treated retinal ECs [72–74]. Increased levels of circulating MALAT1 have also been reported in patients with T2D [75]. Molecularly, in HG-treated ECs, MALAT1 promotes serum amyloid antigen 3, an inflammatory ligand, to increase TNF-α and interleukin-6 (IL-6) [76, 77]. On the other hand, hypoxia also increases MALAT1 expression, while suppression of MALAT1 leads to impaired EC proliferation and blood flow recovery in a mouse model with hindlimb ischemia (HLI) [67, 78]. Interestingly, MALAT1 has been reported to be transcriptionally regulated by Krϋpple-like Factor 4 (KLF4) [79], a key TF responsible for EC homeostasis [80], suggesting a potential of TF in mediating the context-dependent induction of MALAT1. Given these reported roles of MALAT1 in ECs, it will be important to directly assess the role of MALAT1 in the context of DM-PAD, where it may be induced by both HG to promote a pro-inflammatory response, as well as by hypoxia/ischemia to confer a pro-angiogenic response.

*H19* is another lncRNA extensively studied in a number of cell types and biological processes [81, 82]. *H19* is imprinted in a gene cluster containing insulin-like growth factor gene 2 (IGF2) on Chromosome 11 in humans, which is conserved in Chromosome 7 in mice [83, 84]. *H19* is primarily localized in the cytoplasm and plays pleiotropic and cell type dependent roles in ECs, VSMCs, and MΦ during the progression of cardiovascular diseases [85]. Regarding diabetic retinopathy, exposure to high glucose decreases *H19* levels, and this results in the activation of the mitogen-activated protein kinase-extracellular signal-regulated kinase 1/2 (MAPK-ERK1/2) pathway downstream of transforming growth factor beta (TGFβ) signaling, which in turn promotes EndoMT [86]. In line with the requirement of *H19* for EC homeostasis, loss of *H19* in ECs also upregulates inflammatory markers, such as VCAM-1 and intercellular adhesion molecule-1 (ICAM-1), likely through induction of signal transducers and activators of transcription 3 (STAT3) [87]. *H19* is also decreased in the endothelium of aged mice and in the atherosclerotic plaques in humans compared to healthy carotid artery biopsies, suggesting loss of *H19* as part of vascular pathologies. Mechanistically, decreased levels of *H19* led to an upregulation of senescence markers p16 and p21 [88], attendant with a reduction of EC proliferation and inhibition of sprouting capacity of mouse aortic rings [87]. The importance of *H19* is further demonstrated in vivo, with EC-specific inducible *H19* knockout (KO) mice exhibiting increased systolic blood pressure and reduced capillary density in an HLI model [87]. While these studies report a decreased *H19* in ECs in various disease settings, increased circulating H19 has been reported in diabetic patients versus healthy controls [89–91]. These divergent patterns of *H19* in ECs versus circulation are likely due to the levels of *H19* in different cell types and the potential release mechanisms of *H19* into circulating blood, which warrants further investigation. The therapeutic potential of *H19* has also been tested, leveraging extracellular vesicle-mimetic nanovesicles (EMNVs) to deliver *H19* to ECs affected by hyperglycemia [92]. The EMNV-restored *H19* promoted the recovery of diabetic wounds using an in vivo STZ-induced impaired wound closure assay [92]. Given the suppression of *H19* in DM and its protective role in ECs, restoration of *H19* may be a promising approach to attenuate DM-PAD.

Encoded in the *DLK1-MEG3* locus on Chromosome 14q32, maternally expressed gene 3 (*MEG3*) has been extensively studied in cancer [93], diabetes [94], renal ischemia [95], and CVDs [96, 97]. Increased levels of *MEG3* have been reported in cardiomyocytes treated with HG, as well as in serum and villous samples from patients with gestational diabetes, compared to non-diabetic samples [98]. Inhibition of *MEG3* was found to promote the EC angiogenic function in vitro *and* enhance ischemic recovery in aged mice with HLI [99]. The role of *MEG3* in angiogenesis may be through epigenetic regulation by which *MEG3* guides enhancer of zeste homolog 2 (EZH2) to repress integrin subunit alpha 4 (ITGA4) [100]. In line with these findings supporting a negative role of MEG3 in EC homeostasis, overexpression of human *MEG3* promotes aortic cellular senescence and aggravates atherosclerotic lesions in mice [101]. However, *MEG3* has also been shown to protect EC function by regulating the DNA damage response through its interaction with an RNA-binding protein (RBP) polypyrimidine tract binding protein 3 (PTBP3), which restrains the p53-induced EC apoptosis [102]. It is also worthwhile to note that in the context of diabetic retinopathy, MEG3 is decreased in the retinas of STZ-treated mice and HG-treated retinal ECs [103]. *MEG3* knockdown (KD) aggravates retinal vessel dysfunction in vivo, evidenced by increased vascular leakage, increased acellular capillaries, and exacerbated inflammatory damage [102, 104]. Furthermore, *MEG3* has been reported to be increased in plasma samples from patients with diabetes [105, 106]. Taken together, given the complexity of *MEG3-*regulated EC function, the role of *MEG3* in DM-PAD remains to be directly evaluated and identified.

We have identified a lncRNA that enhances eNOS (encoded by *NOS3*) expression (*LEENE*) and regulates EC function [107]. Encoded by *LINC00520* in Chromosome 14 in humans, the transcription of *LEENE* is increased by physiological flow and hypoxia and decreased by pathophysiological conditions, including disturbed flow, HG, and TNF-α [107, 108]. The levels of *LEENE* also decreased in the mesenteric arteries isolated from donors with diabetes. Inhibition of *LEENE* in human ECs decreased the expression of eNOS, Kinase insert Domain Receptor (*KDR*), and a set of genes promoting angiogenesis. Interestingly, the mouse homolog of *LEENE*, also encoded in Chromosome 14, is increased in the atheroprotective region of the aorta as compared to the atheroprone region, as well as in the ischemic limb as compared to the non-ischemic limb in the non-diabetic wild type mice. However, the ischemia induction of mouse *leene* was ablated in the diabetic mice. Furthermore, genetic deletion of *leene* in mice impaired flow perfusion and tissue recovery in the HLI model, especially under diabetic conditions. Importantly, supplementation of human *LEENE* RNA (despite the limited sequence conservation between human and mouse transcripts) rescued the impaired ischemic recovery in the KO mice. Mechanistically, *LEENE* promotes the transcription of pro-angiogenic genes, such as *KDR* and *NOS3*, by interacting with their promoters and RNA binding proteins (e.g., LEO1, a key component of the RNA polymerase II–associated factor complex) [108]. *Leene*-KO mice also developed worsened hypertension in an angiotensin II-infused model [109]. These data support the functional conservation of lncRNAs in humans and mice and highlight the promise of targeting *LEENE* to ameliorate DM-PAD [110]. Of note, *LINC00520* also gives rise to LncRNA Activated by Sheer Stress In the Endothelium (LASSIE), which can interact with intermediate filament protein nestin and the adherens junction components in the cytoplasm to regulate EC permeability [111], suggesting different splicing variants of *LINC00520* may have diverse impacts on EC functions.

*LINC00607*, encoded near *FN1* in Chromosome 2 in the human genome, is another EC-enriched lncRNA found to be unregulated by DM and modulate EC function. KD of *LINC00607* under HG and TNF-α treatment (HT) attenuated pro-inflammatory and pro-EndoMT phenotypes of ECs [112]. Intriguingly, at a baseline condition, *LINC00607* KD caused a profound inhibition of EC angiogenic functions, supporting an essential role of *LINC00607* in maintaining normal EC function [113], which was confirmed by a separate study [114]. Mechanistically, *LINC00607* can interact with a chromatin remodeling protein Brahma-Related Gene-1 (BRG1) to regulate the chromatin state at ERG (ETS Transcription Factor)-binding loci and hence facilitates the transcription of angiogenic genes involved in VEGF-signaling pathway, including *KDR*, Tetraspanin 12 (*TSPAN12*), and Von Willebrand Factor (*VWF*) [114]. These findings suggest a context-dependent mode of action of *LINC00607* in ECs, which may involve complex molecular mechanisms engaging different TFs, co-regulators, and gene targets. For example, under HT, *LINC00607* is upregulated in the nucleus and interacts with the chromatin to increase *FN1* and Serpin Family Member 1 (*SERPINE1*) expression, which may be in concert with the function of MYC [113]. Like *LINC00520* and many other lncRNAs, *LINC00607* produces 13 transcripts that appear to localize to different subcellular compartments. It is possible that different transcripts respond to distinct upstream signals and exert shared or transcript-specific functions, which warrants future investigation. Intriguingly, we found that only the locked nucleic acid (LNA)-GapmeR that inhibited the *LINC00607* level efficiently in the nucleus led to significant *SERPINE1* and *FN1* reduction. In contrast, inhibition of cytoplasmic-localized LINC00607 did not exert the same effect, suggesting a cellular localization-dependent role of *LINC00607* in EC regulation [112]. The functional importance of *LINC00607* and its disease relevance, in particular, to DM-PAD remains to be explored.

## LncRNAs in macrophages (MΦs)

MΦs are involved in both the progression and resolution of PAD. At the steady state, the primary functions of MΦ are tissue surveillance, where cells are poised to fight against infection, remove toxic or dead/dying cells, and restore homeostasis. Under diabetic conditions, such as HT, monocytes, the MΦ precursors, are increasingly recruited to the artery wall. Here, they differentiate into MΦs and secrete factors propagating the chronic low-grade arterial inflammation, which accelerates atherosclerosis, the cause of PAD. Apoptosis of MΦs can also occur during advanced atherosclerosis, contributing to plaque instability and rupture. In the context of PAD, MΦs can be recruited to the ischemic muscle, where they have a continuum of subtypes and polarization states to regulate the inflammatory and immune response [115]. In these contexts, MΦs are dominantly derived from monocytes, and their numbers, particularly pro-inflammatory MΦs, expand greatly in ischemic muscle [116]. Simplistically, MΦs can switch between two phenotypes: M1-like (pro-inflammatory) and M2-like (anti-inflammatory or tissue-repairing), although several other functional and disease-relevant MΦ subtypes have been identified [117–119]. Typically, M1-like MΦs promote inflammatory processes and tissue damage, whereas M2-like MΦs promote tissue repair and resolution of inflammation [120]. These MΦs are involved in the clearance of dead cells and debris from the plaque and support the production of anti-inflammatory cytokines, contributing to tissue healing and stabilization of plaques. In addition, there are resident MΦs seeded during embryonic development, which reside in skeletal muscle. When polarized by M2-ligands, such as interleukin-4 (IL-4), these resident MΦs can promote wound healing and angiogenic repair [121]. Recent studies have revealed unexpected heterogeneity of MΦ and identified new functional subtypes involved in ischemic response. For instance, perivascular MΦs, which accumulate within the ischemic site of human PAD patients and mouse PAD models, produce NO via inducible NOS (iNOS) to regulate blood flow after tissue injury [19].

Although no MΦ-expressing lncRNAs have been directly tested to regulate the development of PAD, lncRNAs have been shown to regulate many functional aspects of MΦs, including chemotaxis, phagocytosis, lipid metabolism, foam cell formation, and efferocytosis [122–124], all of which are highly relevant to DM-PAD. Below, we discuss several lncRNAs that have been shown to regulate MΦ function in DM and atherosclerosis and their potential involvement in DM-PAD.

LncRNA Dynamin 3 opposite strand (*Dnm3os*) is encoded on the opposite strand of Dynamin 3 (*Dnm3*) on Chromosome 1 [125]. Mouse *Dnm3os* and its human ortholog *DNM3OS* share 83% homology. Nuclear-localized *Dnm3os/DNM3OS* are increased by diabetic conditions in monocytes and MΦ. This phenotype was observed in cultured monocytes and MΦ under HG and palmitic acid (PA) treatment, *db/db* mice, high-fat diet-fed mice, and STZ-treated ApoE^−/−^ mice. DNM3OS also increases in CD14^+^ monocytes from patients with DM as compared to healthy controls. Downstream of nuclear factor-κB (NF-κB), *Dnm3os* interacts with nucleolin (NCL), an RBP responsible for regulating chromatin structure, by negatively regulating the expression of pro-inflammatory gene expression through promoting a repressive chromatin state [126]. In diabetic conditions, NCL is decreased while *Dmn3os* is increased, leading to chromatin opening at inflammatory genes. As a result of this dysregulated *Dmn3os*-NCL interaction, inflammation and phagocytosis in MΦ are activated in DM. Given the identified role of *Dnm3os*, *Dnm3os* is also likely to be elevated in DM-PAD and contributes to the unresolved vascular inflammation.

Palmitic Acid-Regulated Anti-Inflammatory lncRNA (*PARAIL)* is another DM-regulated lncRNA found to be altered by DM conditions that regulate MΦ function [127]. *PARAIL (A530072M111Rik*, in mouse) is encoded on Chromosome 8q21.3 and conserved between humans and mice [127]. It is divergently transcribed from its proximal receptor-interacting protein kinase 2 (*RIPK2*), a kinase important for innate and adaptive immunity. Like *DNM3OS*, *PARAIL* is also enriched in the nucleus and increased by PA in CD14^+^ MΦ from healthy donors and THP1-derived MΦs in an NF-κB-dependent fashion. *PARAIL* is highly enriched in the nuclear fractions of MΦs, particularly in the chromatin fraction. *PARAIL* is induced in MΦ during the inflammation resolution phase. Mechanistically, *PARAIL* interacts with an RBP, human antigen R (HuR, gene name ELAV like RNA binding protein 1 (*ELAVL1*)), which is known to bind AU-rich elements (AREs). When *PARAIL* is overexpressed in THP1-derived MΦs, inflammatory markers are decreased. In contrast, when *PARAIL* is suppressed, its interaction with HuR/ELAVL1 increases, promoting the stability and expression of ARE-containing inflammatory genes. In the diabetic *db/db* and STZ-treated ApoE^−/−^ mice, *PARAIL* expression was significantly reduced, supporting that loss of *PARAIL* contributes to enhanced inflammation in a diabetic setting. Interestingly, *PARAIL* was also found to be increased by a pro-inflammatory cytokine IL-1β in human ECs, suggesting its functional role in vasculature in addition to MΦs. However, whether *PARAIL* partakes in the pathogenesis of DM-PAD remains to be determined.

First identified to be upregulated after the occurrence of renal ischemia-reperfusion [128], the Macrophage-Enriched lncRNA Regulates Inflammation, Chemotaxis, and Atherosclerosis LncRNA (*MERRICAL*, previously named AI662270) has been found to play a pro-atherogenic role in mouse models, including ApoE^−/−^ mice fed high-fat diet and Ldlr^−/−^ mice fed a high-fat, high-sucrose diet [128, 129]. *MERRICAL* is encoded in Chromosome 11, proximal to a family of chemokine genes, including C–C Motif Chemokine Ligand 3 (*CCL3*) and *CCL4*. *MERRICAL*, together with *CCL3* and *CCL4*, are co-induced in intimal atherosclerotic lesions of mice. It is also exponentially upregulated with the differentiation of bone marrow-derived macrophages (BMDMs), especially in pro-inflammatory M1-like MΦs. *MERRICAL* is enriched in the nucleus, where it interacts with mixed-lineage leukemia 1 (MLL1), a histone methyltransferase, and an RNA-binding adapter protein WD repeat-containing protein 5 (WDR5) to promote activation of promoter regions of *CCL3* and *CCL4* genes, leading to their increased expression. Consistently, inhibition of *MERRICAL* in vivo decreased leukocyte recruitment to the vascular wall and markedly reduced DM-accelerated atherosclerotic lesion progression. While these findings strongly support a pro-inflammatory and pro-atherosclerotic role of *MERRICAL*, the effect of DM alone on *MERRICAL* and its role in DM-PAD remains to be defined.

Macrophage-Associated Atherosclerosis lncRNA Sequence (*MAARS*), encoded by Gm14461 on Chromosome 2, is a MΦ-specific lncRNA found to localize primarily in the nucleus. *MAARS* is a critical regulator of MΦ apoptosis and efferocytosis, but it has only been studied in mice so far [130]. Its expression dramatically increases in the mouse aortic intima during atherosclerotic progression and decreases with atherosclerotic regression. Inhibition of *MAARS* decreased MΦ apoptosis in vitro and reduced atherosclerotic lesion formation independent of lipid profile and inflammation changes. The anti-atherosclerotic effect of *MAARS* inhibition was attributed to the decrease in MΦ apoptosis and the increase in efferocytosis in the vessel wall. Like *PARAIL*, MAARS also interacts with HuR/ELAVL1, and this interaction prevents cytosolic shuttling of HuR, where it regulates genes involved in apoptosis, such as p53 and caspase-9. The function of *MAARS* in the diabetic and ischemic context remains to be resolved. However, given its relevance in key MΦ functions and possible conservation between humans and mice, it may also play a role in DM-PAD disease progression.

Suppressor of Inflammatory Macrophage Apoptosis LncRNA (*SIMALR)* is a non-conserved, nuclear-localized human lncRNA encoded in Chromosome 6q23.3 [131]. SIMALR directs MΦ polarization, as it is highly expressed in inflammatory/activated M1-like, but not in non-activated anti-inflammatory M2-like MΦ and is increased in the MΦ of human atherosclerotic plaques. In M1-like MΦ, depletion of SIMALR leads to elevated expression of apoptotic markers such as Poly (ADP-ribose) Polymerase (PARP), caspase-9, and caspase-3, suggesting a protective role of SIMALR in MΦs. Mechanistically, SIMALR acts in *trans* by interacting with hypoxia-inducible factor 1α (HIF1α) and promoting the recruitment of this transcription factor to the promoter of Netrin-1 (*NTN1*) a laminin-related protein, which promotes MΦ survival. The SIMALR KD-induced apoptosis was attenuated by treating MΦ with recombinant NTN1 protein [131]. Given the anti-inflammatory and anti-apoptotic effects of SIMALR, it can potentially play a positive role in DM-PAD. Therefore, SIMALR could be a promising target in targeting diabetes-affected MΦs. In line with the study, SIMALR was increased in the MΦs of human atherosclerotic plaques, suggesting an increase of M1-like MΦs, where they can contribute to necrotic core formation [132]. In the context of DM-PAD, SIMALR has not yet been extensively studied. Based on the current data, it is a promising target for mitigating MΦ accumulation and apoptosis in DM-PAD.

Cholesterol homeostasis regulator of microRNA expression (*CHROMR*, previously named *CHROME* [133, 134]) is encoded by AC009948.5 on Chromosome 2q31.2. Like SIMALR, it is not conserved and is only present in the genomes of primates [133]. Expression of *CHROMR* in both the plasma and neointima was increased in patients with atherosclerosis, with the latter being localized to the infiltrating inflammatory cells. Furthermore, its expression was positively correlated with dietary cholesterol levels in MΦs as well as hepatocytes, where it was found to regulate cholesterol efflux. The molecular function of *CHROMR* has been linked to sterol-activated liver X receptors (LXR), key transcription factors known to regulate the response to cholesterol homeostasis. The mode of action of *CHROMR* differs from the aforementioned MΦ lncRNAs. Specifically, *CHROMR* interacts with Argonaute 2 protein (key protein in miRNA-induced silencing complex), and its cellular action on MΦ cholesterol efflux is mediated by sequestering miR-27b, miR-33a, miR-33b, and miR-128 in the cytoplasm [133]. Among these *CHROMR*-associated miRNAs, circulating miR-27b was found to be downregulated in smokers predisposed to PAD [135], while circulating miR-33a was found to be elevated in T2D patient serum [136], and inhibition of miR-33a/b reduced atherosclerosis [137]. Interestingly, *CHROMR* is also increased by viral infection, e.g., Influenza A and SARS-COV-2, for which DM poses significant risks to developing severe thrombotic and inflammatory vasculopathies [133, 138]. Given the crucial role of *CHROMR* in MΦ metabolism, it would be interesting to investigate its regulatory role in DM-PAD.

## LncRNAs in vascular smooth muscle cells (VMSCs)

As the immediate adjacent cell types to ECs, VSMCs and pericytes are mural cells found in the walls of blood vessels. VSMCs receive EC-derived signals, such as NO, cyclic guanosine monophosphate (cGMP), and endothelin-1, and are responsible for vaso-constriction/relaxation to regulate blood flow and vascular tone [139]. While VSMCs constitute the media of the blood vessel wall, pericytes are VSMC counterparts in the capillaries and small venules. Under homeostatic conditions, VSMCs primarily regulate blood vessel diameter and structural integrity in larger vessels, while pericytes are key to the stability and regulation of microvasculature. In a hyperglycemic state, VSMC can undergo proliferation, dedifferentiation, and phenotypic switching, leading to abnormal vascular tone and remodeling, contributing to atherosclerosis and arterial stiffening [16, 140]. On the other hand, the contribution of altered pericyte function in the pathogenesis of PAD is still unclear. However, pericyte dysfunction generally leads to microvascular instability, increased permeability, and impaired angiogenesis, contributing to ischemia and damage in other tissues such as the eye and kidney.

LncRNAs have been shown to regulate various processes contributing to the dysfunction of VSMC and pericytes, as reviewed elsewhere [141, 142]. Some of the lncRNAs discussed earlier in the EC and MΦ sections, e.g., *MALAT1*, *H19*, and *LINC00607*, have also been shown to regulate VSMC function [113, 143, 144]. For example, the EC-enriched *LINC00607* is increased by Angiotensin II (Ang II) (which can be elevated in DM) in VSMCs and likely regulates VSMC proliferation [113]. Thus, the regulatory function of these lncRNAs can act in concert to modulate the pathogenesis of DM-PAD. Below, we summarize several well-characterized VSMC-enriched lncRNAs that regulate VSMC proliferation, phenotypic switching, and migration and discuss their potential contribution to DM-PAD.

Smooth muscle and Endothelial cell–enriched migration/differentiation-associated long NonCoding RNA (*SENCR*) is one of the first lncRNAs identified to be enriched in both VSMCs and ECs encoded by Chromosome 11q24.3 [145]. It is transcribed as an antisense lncRNA from the 5′ end of the Friend leukemia virus integration 1 (*FLI1*) gene, predominantly cytoplasmic, and poorly conserved between human and mouse [145]. SENCR is downregulated in diabetic conditions, such as in *db/db* mice and HG and Ang-II-treated VSMCs [146, 147]. In VSMCs, SENCR KD decreases the expression of myocardin (MYOCD), a master regulator of SMCs contractile genes [148], and increases pro-migratory genes, leading to a hyper-motile SMC phenotype. On the other hand, SENCR overexpression in mice inhibits aortic dissection-associated VSMC proliferation, migration, phenotypic switching, and AngII-induced VSMC apoptosis and ECM degradation [147]. Mechanistically, SENCR regulates VSMC function through miRNAs and Forkhead box O (FoxO) proteins [146, 149, 150]. Interestingly, SENCR is also expressed in ECs and induced by laminar shear stress. By interacting with cytoskeletal-associated protein 4 (CKAP4, a noncanonical RBP) and cadherin 5 (*CDH5*) (encoding vascular endothelial-cadherin (VE-cadherin)), *SENCR* has been shown to maintain membrane integrity and reduce endothelial permeability [151]. Of note is that the levels of SENCR were found to be markedly lower in ECs isolated from patients with premature CAD [152, 153] and in muscles with CTLI [152]. Collectively, these studies indicate SENCR as an essential regulator for vascular homeostasis, whose gain of function may be beneficial in DM-PAD.

As previously mentioned, MYOCD is a key transcriptional cofactor for serum response factor (SRF), which both form a master switch complex controlling VSMC differentiation and lineage [154]. MYOcardin-induced smooth muscle lncRNA inducer of Differentiation (MYOSLID*)* [155], previously annotated *AC007879.7*, is a VSMC-specific antisense lncRNA located on Chromosome 2q33.3 in humans [156]. Despite the proximity to Krϋppel Like Factor 7 (*KLF7)* and cAMP responsive element binding protein 1 (*CREB1)*, MYOSLID does not appear to regulate these neighboring genes. Interestingly, the human *MYOSLID* shares conservation with primates but not lower species, including mice, suggesting an evolutionarily acquired function of *MYOSLID*. MYOSLID is transcriptionally regulated by MYOCD/SRF and localized in the cytosol, where it participates in nuclear localization of megakaryoblastic leukemia 1 (MKL1, another coactivator of SRF and key player in maintaining VSMC differentiation) [157] to promote contractile gene expression governed by MYOCD/SRF, e.g. α-SMA, calponin 1 (CNN1), and myosin heavy chain 11 (MYH11). Thus, MYOSLID acts as a lncRNA amplifier for the maintenance of VSMC differentiation through a feed-forward mechanism.

Cardiac mesoderm enhancer-associated non-coding RNA (*CARMN*), encoded immediately upstream of miR-143 and − 145, two miRNAs regulating VSMC function, is a cardiomyocyte and VSMC-enriched lncRNA conserved in mice and humans located on Chromosome 18 and 5q32, respectively. Originally identified as a regulator of cardiac differentiation [158], the function of CARMN in VSMC has been characterized by three independent studies [159–161]. In high-cholesterol diet-fed atherosclerotic mice, CARMN expression was significantly decreased during the disease progression but restored after returning to a normal diet [160]. Likewise, CARMN expression decreased in human atherosclerotic plaques compared to non-atherosclerotic arteries [160]. In primary human coronary arterial SMCs, CARMN and miR-143 and − 145 were downregulated by proliferative stimuli PDGF-subunit B (PDGF-BB) and oxidized LDL (ox-LDL) [159]. CARMN inhibition, similar to the effect of these stimuli, decreased the contractile markers in VSMC, which appears to be independent of miR-143 and − 145 [159]. The functional importance of CARMN in vivo has been demonstrated using constitutive *CARMN*-KO mice, which exhibited increased lesion area [159], while VSMC-specific and inducible *Carmn*-KO mice had exacerbated neointima formation after vascular injury [161]. Intriguingly, the KD of *CARMN* in atherogenic Ldlr^−/−^mice showed reduced VSMC proliferation and atherosclerotic lesions [160]. These seemingly paradoxical findings suggest a potential dose-dependent effect of *CARMN*, which may also vary between in vitro and in vivo settings. At the molecular level, *CARMN* seems to bind to both MYOCD [161] and SRF [160, 162], which are crucial for VSMC differentiation and contractile gene program [160, 161]. Given the decreased MYOCD in many vasculopathies, it is possible that the MYOCD-interacting lncRNAs, e.g., *MYOSLID and CARMN*, and their impaired actions also play a role in the development of DM-PAD.

While *SENCR*, *MYOSLID*, and *CARMN* are downregulated in conditions that induce VSMC dysfunction, the expression of smooth muscle-induced lncRNA enhancer of replication (SMILR*)* is increased in VSMCs stimulated with IL1-α, and PDGF, two cytokines promoting VSMC-related pathologies [163]. *SMILR* (previously annotated RP11-94a24.1) is encoded downstream of the proximal gene hyaluronic acid synthase 2 (*HAS2*) in Chromosome 8q24.13 in humans. SMILR expression increases in both the cytoplasm and nucleus of VSMCs during phenotypic switching, subsequently leading to its release into the extracellular space, which allows for the detection of SMILR in plasma, making it a potential biomarker for patients at risk of atherosclerosis-related PAD. Mechanistically, SMILR forms RNA-RNA hybrids with centromere protein F, which may subsequently recruit the Staufen1 protein to regulate VSMC proliferation and migration [163]. Given its role as a key driver of VSMC proliferation and its response to proliferation and pro-inflammatory cytokines, which are often increased in DM, one may speculate that SMILR may also partake in the progression of DM-PAD. However, like several lncRNAs discussed earlier, *SMILR* is poorly conserved, making it challenging to interrogate its function in mouse models.

Inflammatory MLK1 Interacting Long Noncoding RNA (INKILN) is another lncRNA recently found to be exclusively expressed in VSMC [164]. INKILN is an intergenic cytoplasmic lncRNA encoded on Chromosome 4q13.3, 20 kb upstream of *IL-8* [164]. INKILN and its interacting protein MKL1 [165] are induced in VSMCs undergoing phenotypic switching, treatment with TNF-α and IL-1β, and in injured carotid arteries. INKILN activates pro-inflammatory gene expression in cultured human VSMCs and ex vivo cultured mouse vessels. However, studying *INKILN* in vivo is not straightforward because, like *MYOSLID*, *SMILR*, and *SENCR*, *INKILN* is poorly conserved between humans and mice. To tackle this challenge, a humanized *INKILN* transgenic (Tg) mouse model was created. After ligation injury, the *INKILN*-Tg mice developed exacerbated neointimal formation in the carotid artery. To explain the underlying molecular mechanism, INKILN was found to interact with and stabilize MKL1 through Ubiquitin Specific Peptidase 10 (UPS10), a deubiquitinating enzyme, leading to MKL1 and p65 transactivation of VSMCs’ pro-inflammatory gene profile [164]. Given the reported pro-inflammatory effect of INKILN in vascular inflammation and VSMC dysfunction, it likely also plays a role in DM-PAD.

**Fig. 1:**
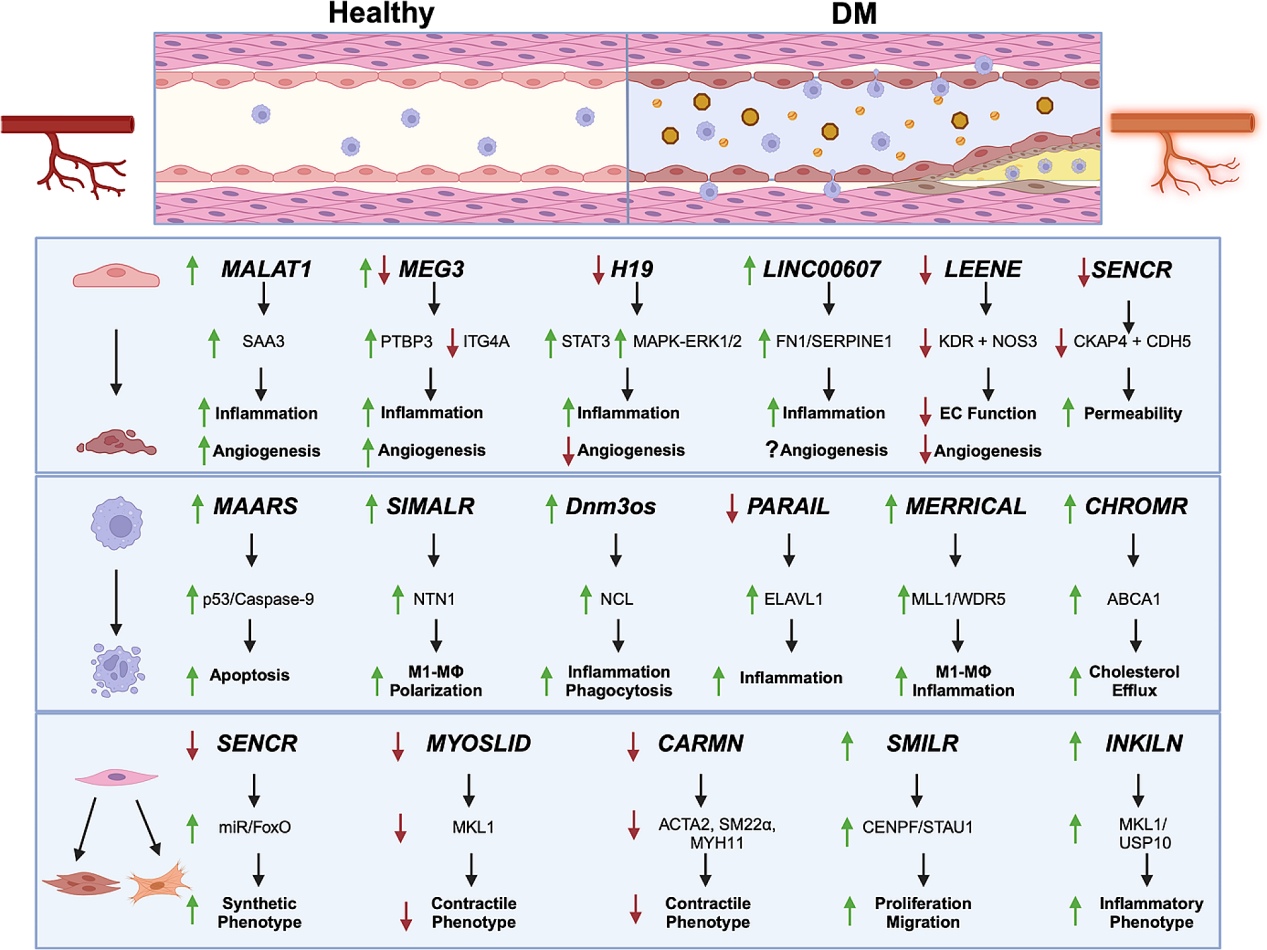
Role of lncRNAs in vascular cell functions related to DM-PAD. A schematic showing the roles of lncRNAs in the functional regulation of endothelial cells (ECs), macrophages (MΦ) and vascular smooth muscle cells (VSMCs) under conditions related to DM-PAD. The reported mechanism of action and their consequent functional role in each cell type is indicated in the boxes. Arrows placed next to each lncRNA and their processes indicate reported or putative changes of expression or activity under DM-PAD.

In addition to the lncRNAs described above (summarized in Fig. 1), a growing list of lncRNAs have been shown to be altered in DM conditions and affect cellular functions relevant to PAD. While some of these lncRNAs are exclusively or preferentially expressed in one or a few cell types, others tend to regulate the functions in multiple cell types with shared or unique modes of action. We have provided a table listing these lncRNAs and their relevant alteration and function to DM-PAD (Table 1).  

**Table 1:** Long noncoding RNAs in diabetes-associated peripheral arterial disease (place before experimental methods and approaches to studying lncRNAs)

Cell types	LncRNA	Human chromosomal position	Species	Cell/tissue type	Disease model	Condition/treatment	Expression change	Targets/regulators/signaling pathways	Function/proposed mechanism	References
Endothelial cells (ECs)	MALAT1	11q13.1	Human	HUVECs	Diabetes, Atherosclerosis, Hindlimb Ischemia (HLI)	High glucose treatment, ox-LDL treatment, hindlimb ischemia by femoral artery ligation of mice	Downregulated under high glucose and ox-LDL conditions	miR-22, ELAVL1, PI3K/Akt, MAPK, NF-κB	Regulates EC proliferation, migration, angiogenesis, inflammation and pyroptosis under diabetic conditions	[67, 78, 166, 167]
	H19	11p15.5	Human	HUVECs	Diabetes, Atherosclerosis	High glucose treatment, ox-LDL treatment	Downregulated under high glucose; Upregulated under Atherosclerosis	Let-7, MAPK, NF-κB	Promotes EC apoptosis and inflammation by regulating Let-7 and activating MAPK and NF-κB signaling pathways	[86, 168, 169]
	MEG3	14q32.2	Human, Mouse, Rat	HUVECs, HRMECs	Diabetes, gestational diabetes mellitus, HLI	High glucose treatment, HLI	Upregulated in blood and villous of patients with gestational diabetes mellitus; Downregulated under high glucose and hypoxic conditions	miR-9, STAT3, p53, TGF-β, Wnt, PI3K/Akt, Notch	Regulates EC proliferation, migration, angiogenesis and apoptosis under diabetic conditions	[98, 103, 104]
	LEENE	14q22.3	Human, Mouse	HUVECs, Mouse primary ECs	Diabetes, HLI, hypertension	High glucose and TNF-α treatment, Hindlimb ischemia model	Downregulated under high glucose and TNF-α condition	LEO1, MYC	Promotes angiogenesis by transcriptionally regulating KDR and NOS3, promotes ischemic recovery	[107–109]
	LINC00607	2q35	Human	HUVECs, HVSMCs	Diabetes	High glucose and TNF-α treatment, Hypoxia	Upregulated under high glucose and TNF-α condition	BRG1, ERG, c-Myc	Regulates angiogenic genes downstream of ERG by recruiting BRG1, promotes angiogenesis	[112–114]
	TUG1	22q12.2	Human	HUVECs	Atherosclerosis	Rapamycin treatment, ox-LDL treatment	Upregulated under rapamycin and ox-LDL treatment	miR-26a, IGF2	Regulates EC apoptosis, proliferation and migration through miR-26a and IGF2	[170–172]
	SENCR	11q24.3	Human	HUVECs, HCAECs, HPAECs	Flow stress	Laminar shear stress	Upregulated in human EC lineages subjected to laminar shear stress	CKAP4, CDH5	Stabilizes adherens junctions by interacting with CKAP4 and CDH5, enhances endothelial barrier function	[151]
	GATA6-AS	18q11.2	Human	HUVECs	Angiogenesis	Hypoxia, TGF-β2 treatment	Upregulated in hypoxic conditions	LOXL2, H3K4me3, TGF-β signaling, PI3K/Akt	Interacts with LOXL2 to regulate endothelial gene expression via histone methylation, controlling endothelial-mesenchymal transition and angiogenesis under hypoxia	[173]
	GAS5	1q25.1	Human, rat	HUVECs, coronary tissues and EC, cardiac microvascular endothelial cells	Atherosclerosis, cardiac microvascular endothelial cells (CMECs) injury	Atherosclerosis model (rat, ApoE^−/−^ mice), homocysteine (HCY)-induced cardiac microvascular endothelial cells (CMECs) injury	Downregulated under diseases	miR-194-3p/TXNIP, ABCA1, miR-33a-5p, miR-193-5p/SRSF10	Regulates endothelial cell functions and atherogenesis by modulating miR-194-3p/TXNIP axis, miR-33a-5p/ABCA1 axis, and miR-193-5p/SRSF10 pathway, influencing oxidative stress, cholesterol homeostasis, and autophagy	[174–176]
	OIP5-AS1	15q15.1	Human	HUVECs	Atherosclerosis	ox-LDL treatment	Upregulated in ox-LDL conditions	GSK-3β, EZH2	Accelerates ox-LDL mediated vascular endothelial cell apoptosis by targeting GSK-3β through recruiting EZH2, leading to reduced GSK-3β expression and promoting apoptosis.	[177]
	NEAT1	11q13.1	Human, mouse	HUVECs, serum	Atherosclerosis	Atherosclerosis model ( ApoE^−/−^ mice), t-BHP-treatment	Upregulated in ApoE^−/−^ mouse and t-BHP-treated HUVECs	miR-181d-5p/CDKN3	Inhibits oxidative stress-induced endothelial cell injury by sponging miR-181d-5p, which upregulates CDKN3, thereby promoting cell viability and reducing apoptosis.	[178]
	FENDRR	16q24.1	Human	Aortic endothelial cells, pulmonary artery endothelial cells	Atherosclerosis, Hypoxic pulmonary hypertension	Ox-LDL treatment, Hypoxia	Downregulated in ox-LDL and hypoxia conditions	miR-18a-5p/PGC-1α, DRP1, m6A RNA methylation	Inhibits ox-LDL induced mitochondrial energy metabolism disorder in aortic endothelial cells via miR-18a-5p/PGC-1α signaling, and regulates hypoxia-induced pulmonary artery endothelial cell pyroptosis by mediating DRP1 DNA methylation through m6A RNA methylation	[179, 180]
	SNHG16	17q25.1	Human	Retinal microvascular endothelial cells (hRMECs)	Diabetic retinopathy	High glucose	Upregulated in high glucose conditions	miR-146a-5p/IRAK1, miR-7-5p/IRS1, NF-κB, PI3K/AKT	Promotes hRMEC dysfunction by sequestering miR-146a-5p and miR-7-5p, acting as a ceRNA to upregulate IRAK1 and IRS1, thus activating NF-κB and PI3K/AKT pathways, facilitating proliferation, migration, and angiogenesis.	[181]
	CA7-4	16q22.1	Human	HUVECs	Diabetic vascular complications	High-glucose (HG) condition	Upregulated in high-glucose conditions	miR-877-3p/CTNNB1, miR-5680/AMPK	Promotes autophagy and apoptosis in high-glucose-induced vascular endothelial cells by sponging miR-877-3p and miR-5680, leading to the regulation of CTNNB1 and AMPK pathways, respectively.	[182]
	NORAD	20q11.23	Human, mouse	HUVECs, ApoE^−/−^ mice	Atherosclerosis (ApoE^−/−^ mice)	Ox-LDL treatment, high-fat diet	Downregulated in ox-LDL and high-fat diet conditions	NF-κB, p53-p21, IL-8, SFPQ	NORAD downregulation promotes ox-LDL-induced endothelial cell injury and atherosclerosis by inducing cell cycle arrest, apoptosis, senescence, and inflammation through NF-κB and p53-p21 signaling pathways, and by interacting with IL-8 repressor SFPQ.	[183]
	p21	6p21.2	Human	Coronary artery endothelial cells (HCAECs)	Atherosclerosis	Ox-LDL treatment	Upregulated in ox-LDL conditions	LOX-1, PKCδ	Mediates ox-LDL-induced apoptosis and expression of LOX-1 in human coronary artery endothelial cells by activating PKCδ. Overexpression of lincRNA-p21 increases ox-LDL-induced apoptosis and LOX-1 expression, while knockdown has the opposite effect.	[184]
	LINC00305	18q22.1	Human	HUVECs	Atherosclerosis	Hypoxia	Upregulated in hypoxia conditions	miR-136	Sponges miR-136 to regulate hypoxia-induced apoptosis in vascular endothelial cells, promoting apoptosis and inhibiting proliferation under hypoxic conditions.	[185]
Macrophages (MΦ)	MAARS	No report	Mouse	Mouse BMDMs, Spleen cells, PBMCs, RAW264.7	Atherosclerosis	Atherosclerosis model (LDLR^−/−^ mice fed with high cholesterol diet)	Increases with atherosclerosis progression, decreases with regression	HuR/ELAVL1	Regulates macrophage apoptosis and efferocytosis by interacting with HuR/ELAVL1, targets: p53, p27, Caspase-9, BCL2	[130]
	PARAIL	8q21.3	Human, Mouse	Human primary monocytes, Mouse BMDMs	Diabetes, Atherosclerosis	Palmitic acid treatment, Atherosclerosis model (STZ-induced diabetic ApoE^−/−^ mice)	Downregulated by palmitic acid treatment	HuR/ELAVL1, NF-κB	Regulates inflammatory genes by interacting with HuR, attenuates macrophage inflammatory response	[127]
	MERRICAL	No report	Mouse	Human primary macrophages, Mouse bone marrow-derived macrophages	Atherosclerosis	Atherosclerosis model (LDLR^−/−^ mice fed with high cholesterol diet). LPS/IL-4 stimulation	Upregulated in intimal lesions of LDLR^−/−^ mice	MLL1, WDR5, H3K4me3	Interacts with MLL1 and WDR5 to upregulate CCL3 and CCL4, promotes macrophage adhesion and atherosclerosis	[129]
	MIST	No report	Human, Mouse	RAW264.7, THP-1 cells, F4/80 + cells from murine stromal fraction	Obesity	High-fat diet-induced obesity model	Downregulated in obesity	PARP1, FABP5	Inhibits M1 polarization by preventing PARP1 binding to inflammatory gene promoters, suppresses inflammation and metabolic dysfunction	[186]
	SIMALR	6q23.3	Human	Human primary macrophages	Inflammation, Atherosclerosis	LPS/IFNγ-induced M1 polarization, human plaque tissue from atherosclerosis patients	Upregulated in M1 macrophages and unstable plaques	NTN1, HIF1α	Regulates macrophage apoptosis and survival in inflammatory conditions	[131]
	DNM3OS	1q24.3	Human, Mouse	Mouse BMDMs, human primary monocytes, RAW 264.7	Diabetes	type 2 diabetic db/db mice, diet-induced insulin-resistant mice and diabetic ApoE^−/−^ mice, high glucose and palmatic acid	Upregulated by high glucose and palmitic acid as well as diabetic mouse models	Nucleolin, NF-κB	Interacts with nucleolin to regulate inflammatory gene expression, promotes inflammation in diabetes	[125]
	PELATON	20q13.13	Human	Human primary macrophages, THP-1 cells	Atherosclerosis	Unstable plaque	Upregulated in unstable plaque	CD36, ROS production	Regulates macrophage functions such as phagocytosis, lipid uptake, and reactive oxygen species production, impacting plaque progression.	[187]
	CHROMR	2q31.2	Human	Human primary macrophages, Hepatocytes	Atherosclerosis	Cholesterol loading, Atherosclerosis model (LDLR^−/−^ mice fed with high cholesterol diet)	Upregulated by cholesterol loading with acetylated LDL	miR-27b, miR-33a/b, miR-128	Regulates macrophage cholesterol efflux through miRNAs, regulates cholesterol homeostasis	[133–135]
	MALAT1	11q13.1	Human	Human primary macrophages, THP-1 cells	Inflammation	LPS treatment	Upregulated by LPS	NF-κB	Regulates inflammatory gene expression by interacting with NF-κB in the nucleus	[188]
	NEAT1	11q13.1	Human	Human primary macrophages, THP-1 cells	Inflammation	LPS treatment	Upregulated by LPS	miR-125a-5p/TRAF6/TAK1	Regulates macrophage polariztion by acting as ceRNA to promote TRAF6 expression	[189]
	Lnc-IL7R	5p13.2	Human	THP-1 cells	Inflammation	LPS treatment	Upregulated by LPS	H3K27me3, IL-7R, E-selectin, VCAM-1, IL-6, IL-8	Regulates the inflammatory response by diminishing LPS-induced expression of inflammatory mediators (E-selectin, VCAM-1, IL-6, IL-8) and modulating histone trimethylation (H3K27me3) at their promoters.	[190]
	THRIL	12q24.31	Human	Human primary macrophages, THP-1 cells	Atherosclerosis	ox-LDL treatment	Upregulated by ox-LDL	FOXO1, ABCA1	Promotes macrophage inflammation and lipid accumulation	[191]
	HOTAIR	12q13.13	Human	Human primary macrophages, THP-1 cells	Inflammation	LPS treatment	Upregulated by LPS	NF-κB	Regulates inflammatory gene expression by interacting with NF-κB and promoting its recruitment to target gene promoters	[192]
	Lnc-MC	19p13.3	Human	Primary macrophages, THP-1 cells	Monocyte/macrophage differentiation	M-CSF-induced differentiation	Upregulated during M2 polarization	miR-199a-5p, ACVR1B	Promotes monocyte/macrophage differentiation by acting as a competing endogenous RNA to sequester miR-199a-5p, thus relieving repression on ACVR1B expression. PU.1 transcriptionally activates lnc-MC and downregulates miR-199a-5p, enhancing differentiation.	[193]
	Kcnq1ot1	11p15.5	Human	Human primary macrophages, THP-1 cells	Atherosclerosis	Western diet, lipid loading	Upregulated in atherosclerosis and lipid-loaded macrophages	miR-452-3p, HDAC3, ABCA1	Promotes lipid accumulation and accelerates atherosclerosis by acting as a competing endogenous RNA (ceRNA) that sponges miR-452-3p, leading to increased HDAC3 expression and decreased ABCA1 expression, which inhibits cholesterol efflux.	[194]
Vascular smooth muscle cells (VSMCs)	SENCR	11q24.3	Human, Mouse	Human aortic VSMCs, Mouse aortic VSMCs	Diabetes, aortic dissection (AD), abdominal aortic aneurysm (AAA), HLI	Ang II treatment, STZ-induced diabetes model, mouse AD, AAA and HLI model	Downregulated in diabetes, CAD and HLI	FoxO1, TRPC6, miR-206/myocardin, miR-4731-5p/FOXO3a	Inhibits VSMC proliferation and migration, maintains VSMC contractile phenotype	[145–147, 149, 150, 152]
	MYOSLID	2q33.3	Human, Rat	Human aortic VSMCs	human arteriovenous fistula (AVF)	overexpression/depletion of MYOSLID	Downregulated expression in veins from failed human arteriovenous fistula and in cultured VSMCs with acquisition of the synthetic phenotype	MYOCD, SRF, SMAD, TGF-β	Downstream effector of MYOCD/SRF and TGFβ1/SMAD pathways, maintains VSMC contractile phenotype	[155]
	CARMN	5q32	Human, Mouse	Human coronary artery SMCs, Mouse aortic SMCs	Atherosclerosis	Atherosclerosis model (LDLR^−/−^ mice fed with high cholesterol diet)	Downregulated in atherosclerotic lesions	MYOCD, SRF, TGF-β	Interacts with MYOCD/SRF to activate transcription of VSMC marker genes, inhibits phenotypic switching	[158–161]
	SMILR	8q24.13	Human	Human saphenous vein SMCs	Atherosclerosis	IL-1α and PDGF treatment	Upregulated by IL-1α and PDGF treatment	HAS2	Promotes VSMC proliferation and migration through HAS2-HA axis, potential biomarker for PAD	[163]
	INKILN	4q13.3	Human, Rat	Human coronary artery SMCs	Atherosclerosis, abdominal aortic aneurysm	Vascular injury model	Downregulated in contractile VSMCs and induced in human atherosclerosis and abdominal aortic aneurysm	MKL1, USP10	Stabilizes MKL1 by inhibiting its ubiquitination, promotes VSMC proliferation and migration	[164]
	GAS5	1q25.1	Human	Human aortic VSMCs	Diabetic PAD, CAD	Carotid balloon injury models, aging	Downregulated in coronary artery disease	p53, P300, SDC1, miR-665	binds to P53 and p300 to suppress the cell cycle and promotes apoptosis, regulates SDC1/miR-665 to regulates senescence of VSMC	[195, 196]
	H19	11p15.5	Human, Rat	Human aortic VSMCs, Rat aortic VSMCs	Arterial calcification	β-glycerophosphate treatment	Upregulated after VSMCs transition	p38 MAPK, ERK1/2, miR-106a-5p/Runx2	promoted VSMCs calcification via the p38 MAPK and ERK1/2 signal transduction pathways and miR-106a-5p/Runx2 axis.	[197, 198]
	FAS-AS1	10q23.31	Human	Human aortic VSMCs	vascular calcification, renal diseases	Hyperphosphatemia treatment	Upregulated in end-stage chronic kidney diseases, renal diseases and vascular calcification	miR-21, FASLG	Silencing FAS-S1 in calcified VSMCs could reduce the content of Ca2+, and alleviate the accumulation of ROS and increasing inflammation cytokines	[199]
	LincRNA-p21	6p21.2	Mouse	Mouse aortic VSMCs	Atherosclerosis model, carotid artery injury model	Atherosclerosis model (ApoE^−/−^mice fed a high-fat diet), carotid artery injury model	Downregulated in atherosclerotic plaques of ApoE^−/−^mice	p53, p300	Inhibits VSMC proliferation and neointimal formation by activating p53	[200]
	RNCR3/LINC00599	8p23.1	Mouse	Mouse aortic VSMCs	Atherosclerosis	Atherosclerosis model, ox-LDL treatment	Upregulated in mouse and human aortic atherosclerotic lesions, and cultured ECs and VSMCs upon ox-LDL treatment	KLF2, miR-185-5p	Forms a feedback loop with KLF2 and miR-185-5p to regulate proliferation and migration	[201]
	BANCR	9q21.11-q21.12	Human	Human aortic VSMCs	Atherosclerosis	patient blood	Upregulated in atherosclerosis	miR-34c, JNK, p38, MAPK	Promotes the proliferation of HASMCs by downregulating miR-34c methylation in atherosclerosis. Promotes VSMC proliferation and migration by activating JNK and p38 MAPK signaling	[202, 203]
	TUG1	22q12.2	Human	Human aortic VSMCs	Atherosclerosis	Atherosclerosis patients and animal model	Upregulated in atherosclerosis	miR-21, PTEN	Promotes VSMC proliferation and migration by regulating miR-21 and PTEN	[204]
	MEG3	14q32.2	Human	Human aortic VSMCs	Atherosclerosis	Atherosclerosis	Downregulated in atherosclerosis	miR-26a/Smad1	Modulates the proliferation/apoptosis balance of VSMCs in atherosclerosis by regulating the miR-26a/Smad1 axis.	[205]
	MALAT1	11q13.1	Human	Human aortic VSMCs	Atherosclerosis	ox-LDL treatment	Downregulated under ox-LDL treatment	miRNA-124-3p/PPARα	Mediates proliferation and apoptosis of VSMCs by sponging miRNA-124-3p to positively regulate PPARα level.	[206]
	CDKN2B-AS1/ANRIL	9p21.3	Human, rat	Human TAD and normal aortic tissues, rat aortic VSMCs	Human thoracic aortic dissection ((TAD))	TAD	Upregulated in TAD	miR-320d, STAT3, miR-126-5p, PTPN7 PI3K-Akt	Works as a molecular sponge for miR-320d and modulates STAT3 expression, binds to miR-126-5p to upregulate PTPN7 and inhibit PI3K-Akt pathway to preventing proliferation and increasing apoptosis	[207–209]
	FOXC2-AS1	16q24.1	Human	Human aortic VSMCs	Atherosclerosis	Atherosclerosis patients, ox-LDL, IL-6, CRP, TNF-α and IL-8 treatment	Upregulated in atherosclerosis	miR-1253/FOXF1	Promotes cell proliferation and inhibited apoptosis via miR-1253/FOXF1 signaling axis	[210]

### Experimental methods and approaches to studying lncRNAs

As reviewed above, numerous lncRNAs have been implicated in a myriad of cellular processes relevant to DM-PAD. To profile lncRNAs, RNA-sequencing (RNA-seq) is widely used to provide quantitative measurements with high sensitivity. In addition, RNA-seq can also be used to identify alternatively spliced transcripts and different isoforms of lncRNAs, which may confer different biological functions. Two commonly used RNA-seq library preparation workflows are polyadenylic acid (polyA) enrichment and ribosomal RNA (rRNA) depletion. PolyA enrichment employs the oligo(dT)-tagged magnetic beads to enrich mRNAs and polyA-containing lncRNAs, thus excluding the non-polyadenylated RNAs [211]. Alternatively, to retain non-polyadenylated RNAs, rRNA can be removed, e.g., by using magnetic beads coupled to oligonucleotides that specifically hybridize with rRNAs [212, 213]. The remaining pool of transcripts preserves the protein-coding mRNAs, lncRNAs, and other non-coding RNAs, providing a favorable tool for *de novo* lncRNA profiling and detecting lncRNA transcripts of low abundance [214, 215]. Given the relatively lower abundance of lncRNAs to mRNAs, a higher sequencing depth is generally recommended.

To allow for more effective and accurate detection of lowly expressed lncRNAs, target enrichment techniques utilizing probe-based hybridization, e.g., RNA CaptureSeq, can be used [216–218]. Target enrichment is critical for full-length or long-read sequencing of individual lncRNA transcripts, e.g., using Oxford Nanopore for direct RNA sequencing [219, 220] or Pacific Biosciences’ Single Molecule, Real-Time sequencing [221, 222]. Following the initial RNA-seq profiling or RNA-seq data mining, it is highly recommended to validate findings from these high-throughput technologies using traditional approaches, e.g., northern blotting and reverse transcription-PCR (RT-PCR), to avoid false positives. In addition, to validate the 5′ and 3′ terminal sequences of lncRNA transcripts, rapid amplification of cDNA ends (RACE) is a valuable assay. Combined with sequencing, also termed RACE-seq, this method effectively annotates the transcription start site and obtains the full-length sequences of lncRNAs [223].

Because LncRNAs are a class of immensely adaptable molecules that function through RNA-DNA, RNA-RNA, or RNA-protein interaction, their biological functions are closely related to their cellular localization. To determine the subcellular localization of lncRNAs, single-molecule fluorescent *in-situ* hybridization (smFISH) and fractionation are two standard and complementary methods. smFISH utilizes the amplification of multiple tandem probes that can bind to their RNA targets where their fluorescent signal is enhanced, which allows visualization of low-abundance RNA targets [224, 225]. smFISH enables the quantification of RNA transcripts by molecular counts and the detection of co-localization of multiple lncRNAs. As expected, the key to reliable smFISH detection is the rigorous probe design, which can be facilitated by bioinformatic tools such as ProbeDealer [226]. Due to the low abundance and presence of repetitive elements in some lncRNAs, off-target effects, and microscopy interference such as autofluorescence, lncRNA detection by smFISH could be challenging or infeasible in some cases. Cell fractionation, which physically isolates subcellular compartments based on intact organelle purification or partition along sucrose gradients, can be applied to overcome the challenges of smFISH [227, 228], which can also provide validation for smFISH. RNA extracted from different subcellular fractions can be quantified by RT-PCR for individual lncRNAs or RNA-seq for the entire transcriptome. With this method, lncRNAs have been mapped to chromatin-associated fraction, nuclear soluble fraction, and cytoplasmic fraction.

Recent developments of new techniques such as engineered ascorbate peroxidase (APEX)-catalyzed proximity biotinylation and multiplexed error robust fluorescence in situ hybridization (MERIFISH) have provided more powerful tools for improved lncRNA profiling. Originally utilized as a genetic tag for protein mapping, APEX catalyzes the formation of biotin-phenoxyl radicals between its substrates, biotin-phenol, and hydrogen peroxide in different compartments of live cells and covalently biotinylated nearby endogenous proteins. By cross-linking the protein-RNA in situ, APEX-tagged subcellular proteomes also provide localization information of their binding RNA partners [229, 230]. When combined with RNA Immunoprecipitation, these RNAs can be identified by qPCR or profiled by RNA-seq. APEX-RNA-seq has been shown to identify hundreds of cytoplasmic and nuclear lncRNAs in HEK-293T cells. As a high-throughput version of smFISH, MERFISH was developed to simultaneously detect hundreds of RNA transcripts using combinatorial FISH labeling with encoding schemes in successive rounds of hybridization and imaging [231, 232]. However, extensive optimization is needed to apply MERFISH to detect multiple lncRNAs within different subcellular compartments, esp. in the nucleus. As each of these techniques has strengths and limitations, one should consider using at least two independent methods to confirm the subcellular localization of a lncRNA candidate.

As many lncRNAs exert their activity via RNA-protein interactions, various modern approaches have increased the capacity to identify their protein partners for investigating lncRNA molecular mechanisms and pathways. Cross-linking and immunoprecipitation (CLIP) or RBP immunoprecipitation (RIP) can be employed to examine if the given lncRNA is binding to a protein of interest [233, 234]. Multiple techniques have also been developed to capture lncRNA-chromatin interaction in either one vs. the whole genome or all RNAs vs. the whole genome manner [235], among which the chromatin isolation by RNA purification (ChIRP) is the most cited method and has been used to identify genomic sites and RBPs bound by many lncRNAs [236, 237].

To investigate the cellular functions of lncRNAs, experiments that alter RNA function are commonly used to isolate one variable. The gain-of-function of lncRNA function typically uses viral vectors, such as adenovirus, lentivirus, and adeno-associated virus. For example, we have used adenovirus to overexpress human LEENE RNA in the mouse ablated with the *leene* homolog, which improved ischemic recovery [108]. To study the loss-of-function of lncRNAs, the most common techniques used are RNA interference (RNAi) and antisense oligos (ASOs). While RNAi is mainly active in the cytoplasm and thus more suitable to target cytoplasmic lncRNAs, ASO, esp. the LNA-GapmeRs, can induce the RNase H-dependent RNA degradation and therefore inhibit the nuclear-localized lncRNAs [238–241], as demonstrated by many studies including the ones cited in this article. Besides these perturbations at the RNA levels, various clustered, regularly interspaced short palindromic repeats (CRISPR) systems are powerful tools for both gain and loss-of-function studies. CRISPR interference (CRISPRi) and activation (CRISPRa) facilitate efficient control over lncRNA transcription inhibition or activation by coupling nuclease-deficient Cas9 (dCas9) with a transcriptional repressor or activator, such as Krüppel associated box (KRAB) or VP64, respectively [242]. In addition, to tether a specific lncRNA to specific DNA loci, CRISPR-DISPLAY (CRISP-DISP) can be employed, which uses dCas9 to deploy RNA cargos to DNA loci as directed by guide RNA [243, 244].

**Fig. 2:**
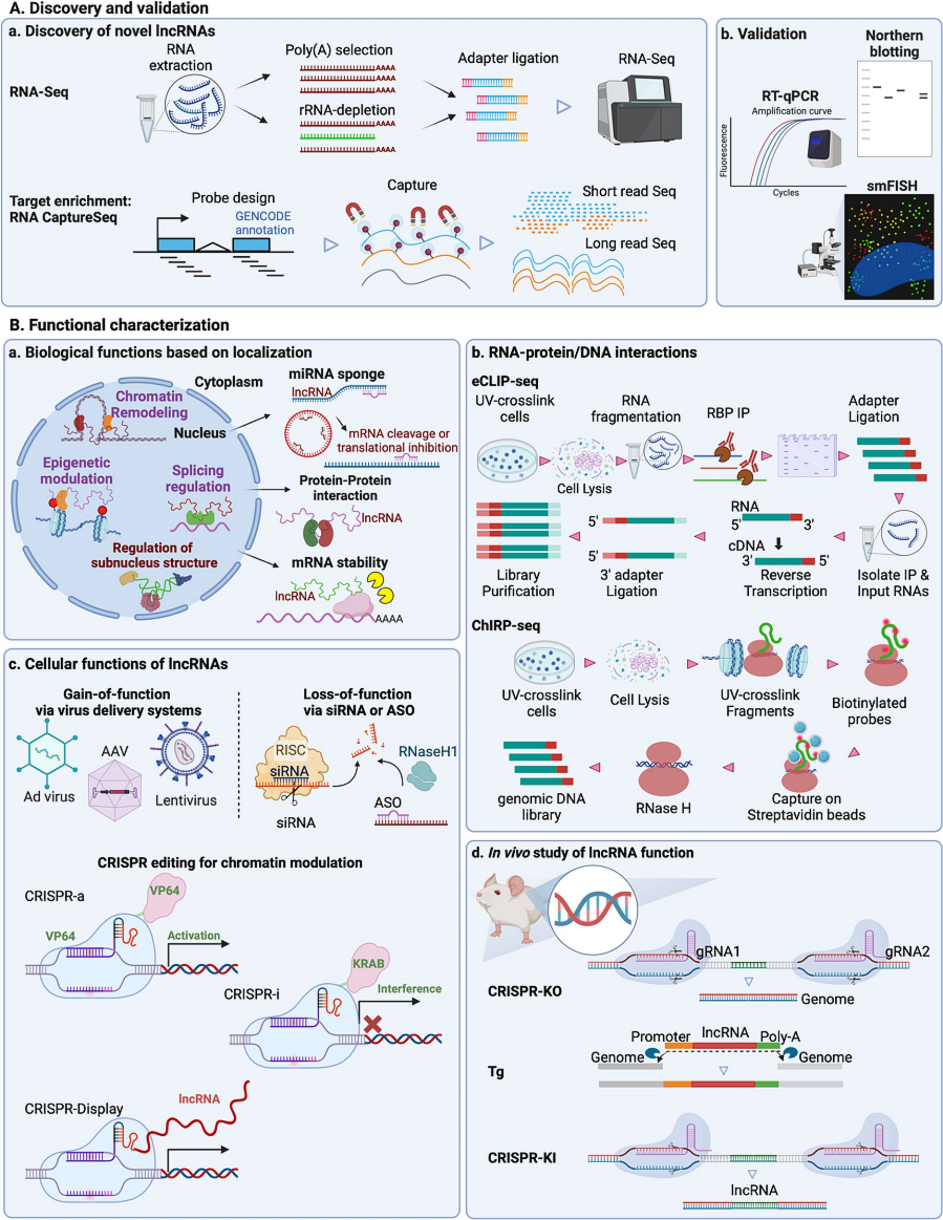
Experimental methods and approaches for lncRNA study. **A** Discovery and validation of lncRNAs using **a** RNA-seq and CaptureSeq and **b** using Northern blotting, RT-qPCR, and smFISH. **B** Characterization of lncRNA function. **a** Depending on their subcellular localization, lncRNAs mediate miRNA sponging, protein-protein interactions, or mRNA stability in the cytoplasm or epigenetic regulation, chromatin state modulation, or RNA splicing in the nucleus. **b** RNA interactions with proteins or DNA can be identified through eCLIP- and ChIRP-seq. **c** Investigation of the role of lncRNAs in cells through gain-of-function using of Ad, AAV, and lentivirus or loss-of-function via siRNA or ASO. CRISPRa, CRISPRi, and CRISPR-Display can be used to reveal chromatin-associated lncRNA functions. **d** In vivo studies using mouse models with CRISPR-KO, Tg, and CRISPR-KI of lncRNA of interest. *smFISH* single-molecule fluorescence in situ hybridization, *eCLIP*: enhanced crosslinking and immunoprecipitation, *ChIRP* chromatin isolation by RNA pulldown, *Ad* adenovirus, *AAV* adeno-associated virus, *ASO* antisense oligonucleotides, *RISC* RNA-induced silencing complex, *CRISPRa/i* CRISPR activation/interference, *gRNA* guide RNA, *CRISPR-KO* CRISPR-knockout, *Tg* transgenic,* CRISPR-KI* CRISPR-Knockin

To investigate the in vivo function of lncRNAs, one can employ a similar approach as used for protein-coding genes, most typically genetic deletion in mice. However, one needs to be cautioned that deletion of lncRNAs derived from regulatory genomic regions, e.g., enhancers, can ablate the function of DNA rather than RNA. One approach to address this is to re-introduce the lncRNA in conditions where DNA is removed, e.g., as demonstrated for *LEENE* [108]. Another approach to address this and the low lncRNA homology [245] is to create a knock-in (KI) of human lncRNA expression in mice to study the exogenous expression of human lncRNAs under the in vivo setting, as demonstrated for the VSMC-specific *INKILN* [164]. A KI lncRNA mouse model is generated by targeted insertion of the lncRNA gene or cDNA at a selected locus, most commonly at the genomic locus homologous to humans. DNA without any functional gene or regulatory elements flanks the insert, and homologous recombination allows the target of the *trans*-gene to that specific integration site. These genetic engineering strategies can be combined with tissue-specific targeting for protein-coding gene studies. Alternatively, gain- or loss-of-function of lncRNA may be achieved by using viral vectors or ASOs as introduced for cell-based studies, using careful consideration of the administration route based on the lncRNA features and research aims. Interested readers are recommended to read several reviews discussing the utilization of mouse models to study lncRNAs [246–249]. Upon obtaining the relevant mouse models, one can subject them to relevant models of diabetes(e.g., either STZ-induced T1D or special diet-induced T2D) or atherosclerosis (e.g., through induction of proprotein convertase subtilisin/kexin type 9 (PCSK9) under a high-cholesterol diet), which can be further combined with the femoral artery ligation to induce HLI, the most commonly used model for PAD [68, 250–253]. We have provided a graphical summary of methods to discover, validate, and functionally characterize lncRNAs above (Fig. 2).

## Conclusion and perspectives

LncRNAs have emerged as crucial regulators of various cellular processes, including those in several vascular cell types discussed herein. These RNA molecules, which do not encode proteins, influence gene expression and cellular function through diverse mechanisms such as chromatin remodeling, transcriptional control, and post-transcriptional regulation. In the context of vascular cells, lncRNAs play significant roles in endothelial function, smooth muscle cell behavior, and the inflammatory responses critical to vascular health. Importantly, in DM-PAD, a condition characterized by reduced blood flow and vascular complications due to DM, lncRNAs have been implicated in disease progression and pathogenesis. Altered lncRNA expression in diabetic conditions can exacerbate vascular dysfunction, contributing to the development and progression of PAD.

While most of the lncRNA studies focused on their intracellular function, recent studies have revealed the presence of lncRNAs in circulation, either in a cell-free format or encapsulated in extracellular vesicles [254–256], hinting at a potential role of lncRNA in cell communication and its potential as biomarkers for a disease state. At the time of this review, no publication has reported any systemic lncRNA profiling in circulating peripheral blood monocytes, serum, or plasma samples of patients with PAD. However, as discussed earlier, several lncRNAs, e.g., H19, have shown diagnostic potential in CAD [89, 257–259]. It will be of interest to explore the diagnostic value of lncRNAs in PAD and DM-PAD.

Targeting lncRNAs represents an innovative therapeutic strategy for DM-PAD. Compared to protein-coding genes, their non-coding feature, relatively low expression levels, and high tissue/cell type-specificity may provide higher specificity and lower off-target effect with a lower necessary dosage [27]. Emerging evidence supports the idea that the impaired angiogenic signaling pathways downstream of the angiogenic growth factor can be governed by lncRNAs [47]. Therefore, modulating the levels of these lncRNAs can likely yield additive therapeutic effects through cooperative mechanisms. The most developed approach to targeting lncRNAs is ASO-based therapeutics [260, 261]. Several ASOs targeting oncogenic lncRNAs are currently being tested clinically [262, 263], which will yield valuable information for the therapeutic potential of targeting lncRNAs for non-cancerous diseases such as DM-PAD. Complementary to inhibiting the disease-driving lncRNA, another approach would be to restore the DM-impaired lncRNAs that would otherwise confer anti-inflammatory and pro-repair effects. This may be achieved by viral vectors or modified RNAs, which have been tested in clinical trials for PAD therapeutics [264] and proven successful in the vaccines against SARS-CoV2 [265–268].

In conclusion, the accumulating evidence supports a highly complex and integrative lncRNA-mediated regulation of various cell types driving DM-PAD. The dysregulated lncRNAs in DM can impair vascular homeostasis and regenerative capacity and provoke prolonged vascular inflammation, remodeling, and damage. Beyond what has been discussed in this review, lncRNA dysregulation in other cell types, such as pericytes and fibro/adipogenic progenitors, may also exacerbate vascular dysfunction [269–271]. Improved understanding of the intricate molecular mechanisms governing context-dependent lncRNA functionality across various cell types and careful evaluation of their diagnostic and therapeutic potential in DM-PAD presents a new opportunity to address this challenging medical condition.

## Data Availability

No datasets were generated or analysed during the current study.

## Abbreviations

Ang II: Angiotensin II
APEX: Ascorbate peroxidase
AREs: AU-rich elements
ASOs: Antisense oligos
BMDMs: Marrow-derived macrophages
BRG1: Brahma-related gene-1
CAD: Coronary artery disease
cAMP responsive element binding protein 1: Camp responsive element binding protein 1
CARMN: Cardiac mesoderm enhancer-associated non-coding RNA
CCL: C-C Motif Chemokine Ligand
CDH5: Cadherin 5
cGMP: Cyclic guanosine monophosphate
ChIRP: Chromatin isolation by RNA purification
CHROMR/CHROME: Cholesterol homeostasis regulator of microRNA expression
CKAP4: Cytoskeletal-associated protein 4
CLIP: Cross-linking and immunoprecipitation
CLTI: Critical limb tissue ischemia
CNN1: Calponin 1
CRISP-DISP: CRISPR-DISPLAY
CRISPR: clustered regularly interspaced short palindromic repeats
CRISPRa: CRISPR activation
CRISPRi: CRISPR interference
CVD: cardiovascular diseases
DM: Diabetes mellitus
dCas9: deficient Cas9
Dnm3: Dynamin 3
Dnm3os: LncRNA Dynamin 3 opposite strand
ECM: extracellular matrix
ECs: endothelial cells
ELAVL1: ELAV like RNA binding protein 1
EMNVs: extracellular vesicle-mimetic nanovesicles
EndoMT: endothelial-mesenchymal transition
eNOS: endothelial NO synthase
FLI1: Friend leukemia virus integration 1
FN1: fibronectin 1
FoxO: Forkhead box O
HAS2: hyaluronic acid synthase 2
HG: high-glucose
HIF1α: hypoxia-inducible factor 1α
HLI: hindlimb ischemia
HT: HG and TNF-α treatment
HuR: human antigen R
